# Epithelial cell specific Raptor is required for initiation of type 2 mucosal immunity in small intestine

**DOI:** 10.1038/s41598-017-06070-w

**Published:** 2017-07-17

**Authors:** Bola Aladegbami, Lauren Barron, James Bao, Jason Colasanti, Christopher R. Erwin, Brad W. Warner, Jun Guo

**Affiliations:** 1 Division of Pediatric Surgery, St Louis Children’s Hospital, Department of Surgery, Washington University School of Medicine, St. Louis, MO 63110 USA; 2 Department of Biology, Washington University in St. Louis, St. Louis, MO 63110 USA; 3 Fischell Department of Bioengineering in the A. James Clark School of Engineering at the University of Maryland, College Park, MD 20742 USA

## Abstract

Intestinal tuft cells are one of 4 secretory cell linages in the small intestine and the source of IL-25, a critical initiator of the type 2 immune response to parasite infection. When Raptor, a critical scaffold protein for mammalian target of rapamycin complex 1 (mTORC1), was acutely deleted in intestinal epithelium via Tamoxifen injection in *Tritrichomonas muris* (Tm) infected mice, tuft cells, IL-25 in epithelium and IL-13 in the mesenchyme were significantly reduced, but Tm burden was not affected. When Tm infected mice were treated with rapamycin, DCLK1 and IL-25 expression in enterocytes and IL-13 expression in mesenchyme were diminished. After massive small bowel resection, tuft cells and Tm were diminished due to the diet used postoperatively. The elimination of Tm and subsequent re-infection of mice with Tm led to type 2 immune response only in WT, but Tm colonization in both WT and Raptor deficient mice. When intestinal organoids were stimulated with IL-4, tuft cells and IL-25 were induced in both WT and Raptor deficient organoids. In summary, our study reveals that enterocyte specific Raptor is required for initiating a type 2 immune response which appears to function through the regulation of mTORC1 activity.

## Introduction

Epithelial tuft cells were first described over 60 years ago in respiratory and gastrointestinal cells^1^. In the small intestine, tuft cells represent less than 1% of the cell population and belong to one of the four intestinal secretory cell linages. This cell type was originally characterized by the long, blunt microvilli protruding into the lumen and by its unique tubulovesicular system^1^. The transient receptor potential cation channel, subfamily M, member 5 (Trpm5), a taste receptor, is surprisingly also found in tuft cells^2^. The generation of a *Trpm-5 -eGFP* reporter mouse line resulted in most of the known tuft cell markers in intestine, such as Sox-9, COX-1 and COX-2^2^. Doublecortin-like kinase 1 protein (DCLK1) was initially considered a marker for putative quiescent cancer stem cell in intestine^3, 4^, but was later found to be a marker of tuft cells and is used extensively for tuft cell identification.

The function of tuft cells was revealed recently by three simultaneous studies^5–7^. Tuft cells were shown to work as mediators for the intestinal type 2 immune response. When the intestine is infected with parasites, tuft cells respond by increasing IL-25 expression. IL-25 stimulates group 2 innate lymphoid cells (ILC2) in the lamina propria to produce IL-13. IL-13 stimulates intestinal stem cells or transient-amplifying cells to differentiate into greater numbers of tuft cells and goblet cells creating a positive feedback loop until all parasites are effectively expelled from the intestine^8–10^.

The exact mechanism by which tuft cells sense the signal from parasites to coordinate type 2 immune response is still unknown. However, studies have shown that taste receptor Trpm 5 in tuft cells is critical in initiating the response^6^. The transcription factor pou2f3 also seems to be important for tuft cell lineage specification in response to parasitic infection^5^. Other proteins such as Stat 6, IL-4R, and IL-2R have also been shown to play important roles in this response^6^.

Raptor is a component of the mTORC1 complex in addition to mLST8/GβL, PRAS40, deptor and mTOR^11, 12^. mTORC1 is a well-known mediator of nutrition-sensitive regulation of cell proliferation and cell growth. Raptor is required for mTORC1 signaling in small intestinal epithelial cells. However, Raptor does not affect crypt cell proliferation at baseline nor under the injury model of small bowel resection^13^. But, Raptor does affect secretory cell linage differentiation, as we and others have demonstrated that diminished Raptor expression resulted in significantly reduced Paneth cell and goblet cell abundance, and increased enteroendocrine cell population^13, 14^.

Much of the function regarding tuft cells remains unclear as they are a newly established member of the secretory cell lineage. Our aim was to determine whether Raptor plays a role in the differentiation of tuft cells. Surprisingly, we found Raptor knockout in intestinal epithelial cells blunted tuft cell function and type 2 immune response to Tm infection.

## Methods

### Mice

All protocols and experiments were approved by the Washington University Animals Studies Committee (Protocol #20150285) and followed National Institutes of Health (NIH) animal care guidelines. C57BL/6 mice, *Raptor (flox/flox)* mice were purchased from the Jackson laboratory (Bar Harbor, ME). *Villin-Cre* ^*ER*^ mice were obtained via a generous donation from Sylvie Robine (Curie Institute, Paris, France). Intestinal epithelial-specific Raptor knockout mice were generated by crossing *Villin-Cre* ^*ER*^ mice with *Raptor (flox/flox)*. Wild type littermates *Villin-Cre* ^*ER*^ (−); *Raptor (flox/flox)* were used as control mice. Mutant mice were maintained on a C57BL/6 background. Tamoxifen (TAM) (Sigma, St. Louis MO) was dissolved in sunflower oil at 10 mg/ml, injected intraperitoneally at 50 µg/per gram body weight for 3 consecutive days to induce deletion of gene expression. Rapamycin (LC laboratories, Woburn MA) was dissolved at 10 mg/mL in 100% ethanol, diluted in vehicle (5% Tween-80, 5% PEG-400 with PBS) and injected intraperitoneally at 4 mg/kg body weight. Mice were kept in the animal holding area with a 12-hour light-dark cycle and given rodent chow ad lib after weaning.

### Small bowel resection

The 50% proximal SBR procedure has been previously described^15^. The intestinal resections were performed by transecting the bowel 1 to 2 cm distal from the ligament of Treitz and at 12 cm proximal to the ileocecal junction followed by removal of the intervening segment. Intestinal continuity was re-established by an end-to-end primary anastomosis using interrupted 9-0 monofilament sutures. After the operation, mice were provided free access to water for the first 24 h after surgery. Mice were then fed with a standard liquid diet (PMI Micro-Stabilized Rodent Liquid Diet LD 101; Test Diet, Richmond IN) until sacrifice.

### Tissue harvest

At the time of sacrifice, a midline laparotomy was performed and the entire small intestine was flushed with ice-cold phosphate buffered saline containing protease inhibitors (0.2 nM phenylmethysulfonyl fluoride, 5 μg/mL aprotinin, 1 μM benzamidine, 1 mM sodium orthovanadate, and 2 μM cantharidin). A 2-cm segment of ileum small bowel was fixed in 10% neutral-buffered formalin for histology. Crypt, villus, and mesenchyme were isolated as we have previously described^16^. Protein extracted from isolated enterocytes was used for Western blot assay, while RNA extracted from the epithelium and mesenchyme were used for real-time PCR assays.

### Histology, immunohistochemistry and organoid whole-mount immunofluorescence

Intestinal tissue was paraffin-embedded and cut into five-micron-thick longitudinal sections followed by H&E staining. For immunohistochemistry, the un-stained slides were deparaffinized and blocked with 3% hydrogen peroxide in methanol. Antigen retrieval was performed using Diva Decloaking solution (Biocare Medical, Concord CA) (120 °C for 2 min using a pressure cooker). Slides were blocked with avidin-pink and biotin-blue (Biocare Medical), treated with anti-DCLK1 antibody (#62257, 1:400; Cell Signaling Technology, Danvers MA) in DaVinci Green (Biocare Medical), incubated overnight at 4 °C, and visualized with biotinylated goat anti-rabbit IgG (Jackson ImmunoResearch Inc. West Grove PA) followed by streptavidin-horseradish peroxidase (Jackson ImmunoResearch Inc. West Grove PA), diaminobenzidine (Sigma-Aldrich), and hematoxylin counterstaining. Organoid whole-mount immunofluorescence staining was performed according to published method^17^ with minor modification. Briefly, the organoid was fixed with 10% neutral-buffered formalin, quenched with 50 mM NH_4_Cl, permeabilized with 0.5% Triton X-100, blocked with 5% BSA, treated with DCLK1 antibody (#62257, 1:100; Cell Signaling Technology, Danvers MA) and finally visualized with Alexa Fluor^®^ 488 Conjugate anti-rabbit secondary antibody (#4412, 1:250; Cell Signaling Technology, Danvers MA).

### Western blotting

Isolated enterocytes were lysed with sodium dodecyl sulfate sample buffer (50 mM Tris-HCl pH 6.8, 2% sodium dodecyl sulfate, 10% glycerol, and 5% mercaptoethanol). The lysate was then heated for 5 min at 100 °C, and the protein concentration was determined using the RC-DC kit (Bio-Rad, Hercules CA). Proteins were loaded in equal amounts for Western blotting. Antibodies used in this study were Raptor (#2280), p-S6K (#9234), pS6 (S240/244) (#5364), DCLK1 (#62257), Stat 6 (#5397), GAPDH (#5174) (all from Cell Signaling Technology, Danvers MA) and p-Stat 6 (sc-11762, Santa Cruz Biotechnology, Dallas Texas). The proteins were detected using Bio-Rad ChemiDocTM XRS + system with image Lab TM software (Bio-Rad, Hercules CA).

### Real-time quantitative PCR

Isolated enterocytes were homogenized in lysis buffer and RNA was extracted according to the manufacturer’s protocol (RNAqueous kit; Ambion, Austin TX). Total RNA concentration was determined using a NanoDrop Spectrophotometer (ND-1000; NanoDrop Technologies, Wilmington DE). Primers for IL-25, IL-13, TSLP, IL-4, IL-5 and DCLK1 were obtained from Life Technologies (Carlsbad, CA). β-Actin was used as the endogenous control (Applied Biosystems, Foster City CA). Applied Biosystems 7500 Fast Real-Time PCR system was used to obtain relative RNA expression using one-step TaqMan technology. All RT-PCR results are normalized to the β-actin endogenous control.

### Stool DNA extraction and Tm detection by real-time PCR

DNA was extracted from fecal samples per the manufacturer’s protocol for the QIAamp DNA stool mini kit (Qiagen, Germantown, MD). Quantitative PCR (qPCR) was performed using KAPPA SYBR fast Universal PCR (KAPPA Biosystems). Tritrichomonas muris was detected by primers 5′-GCTTTTGCAAGCTAGGTCCC-3′ and 5′-TTTCTGATGGGGCGTACCAC-3′ which specifically recognize 28S rRNA gene^6^. Total bacteria load was detected by primers 5′-TCCTACGGGAGGCAGCAGT-3′ and 5′-GGACTACCAGGGTATCTAATCCTGTT-3′ which recognize broad range of bacteria 16S rRNA gene^18^. PCR was done using Applied Biosystem 7500 PCR system. The Tm burden in stool was calculated using the following equation (2^−ΔCt(28S-16S)^) as described by Dr. Garret’s group^6^.

### *Ex vivo* enterocytes and Organoid stimulation

The small intestine was flushed with ice-cold phosphate buffered saline and cut into 1 cm segments and then transferred into BSS buffer (1.5 mM KCl, 96 mM NaCl, 27 mM sodium citrate, 8 mM KH_2_PO4, 5.6 mM Na_2_HPO4 and 15 mM EDTA) (isolation buffer), vortexed at 4 °C for 10 minutes, transferred into fresh isolation buffer, and vortexed for another 10 minutes. The crypts and villi were separated via a 70 µm cell strainer, spun down at 500 g for 3 minutes and washed once with ice-cold PBS. For *ex vivo* stimulation, the enterocytes were resuspended in DMEM/F12 medium and stimulated with 50 ng/ml IL-4 (#5208, Cell Signaling Technology, Danvers MA) or IL-13 (#210-13, PEPROTECH, Rocky Hill NJ) in a tissue culture incubator for 10 minutes. The enterocytes were collected, lysed with sodium dodecyl sulfate sample buffer and analyzed by Western blot assay. For organoid culture, the crypt fraction was mixed with matrigel (Corning® Matrigel® Growth Factor Reduced (GFR) Basement Membrane Matrix, #356231, Corning NY) and the standard protocol^17^ was followed to generate organoids using IntestiCult™ Organoid Growth Medium (Mouse) (#06005, STEMCELL Technology, Cambridge MA). To induce tuft cell differentiation, the organoids were stimulated with 50 ng/ml IL-4 for at least 48 hours. The organoids were then fixed for immunofluorescence, or harvested for protein & mRNA analysis.

### Statistical analysis

All values are reported as mean ± standard deviation of the mean. Statistical analysis was performed using Student’s t-test to compare the two experimental groups. A p-value of less than 0.05 was considered significant.

## Results

### Raptor is required for maintaining the type 2 immune response to Tm infection

**Figure 1:**
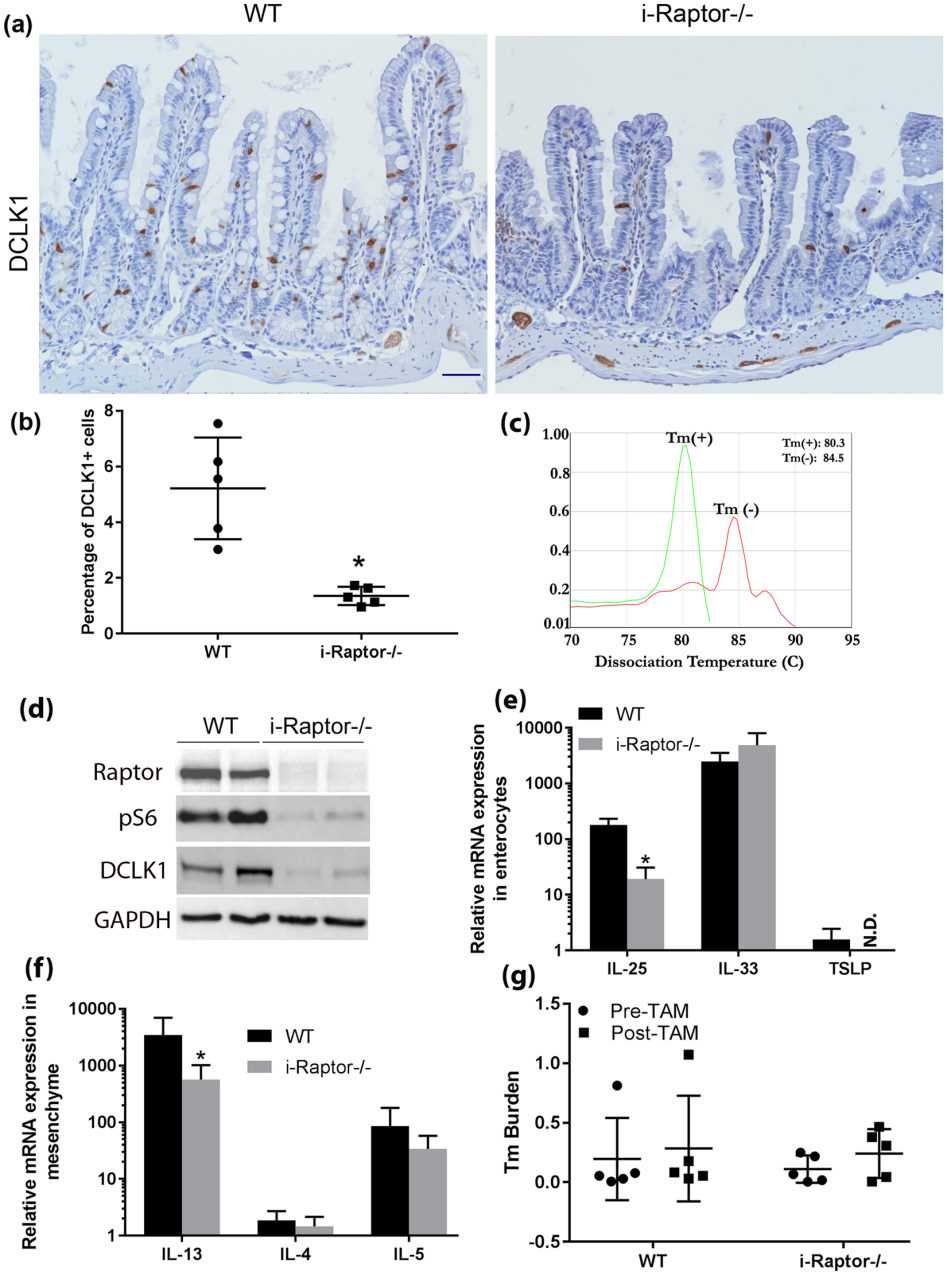
Enterocyte-specific Raptor is required for maintaining tuft cell abundance and function. *Vilin-Cre* ^*ER*^ (−); *Raptor* (*f*/*f*) (WT, n = 6) and *Vilin-Cre* ^*ER*^ (+); *Raptor* (*f*/*f*) (*i*-*Raptor*−/−, n = 6) mice were injected intraperitoneally with Tamoxifen (TAM) for 3 consecutive days and small intestine removed 2 weeks later. (**a**) Representative immunohistochemistry (IHC) shows tuft cells in brown using anti-DCLK1 antibody. (**b**) Quantification of tuft cells based on at least 20 well orientated crypt-villi axis. (**c**) Representative dissociation curve shows RT-PCR amplification of 28S rRNA gene from Tritrichomonas muris (Tm) positive or negative feces. (**d**) Representative Western blot analysis of the protein expression as indicated. GAPDH was used as a loading control. (**e**) Real-time PCR measurements of cytokines expression in enterocytes as indicated. (**f**) Real-time PCR measurements of cytokines expression in lamina propria as indicated. (**g**) Tm burden quantified from stools collected before and 7 days after TAM injection. Mean ± SD is shown. Student’s t test (*p < 0.05).

To determine the role of mTORC1 in regulating tuft cell differentiation via acute disruption of Raptor expression in intestinal epithelial cells, we bred *Villin-Cre* ^*ER*^ mice with *Raptor (flox/flox)* mice to generate *Villin-Cre* ^*ER*^ (−); *Raptor(f/f)* (*WT*) and *Villin-Cre* ^*ER*^ (+); *Raptor(f/f)* (*i-Raptor−/−*) mice. Surprisingly, when we stained *WT* small intestinal tissue with DCLK1 antibody looking for tuft cell differentiation, we found a much higher percentage of tuft cells (5.2%) in the *WT* line (Fig. 1a,b). The tuft cell population in small intestine is normally under 1%^1^. With the recent studies having shown that tuft cells are critical for initiating a type 2 immune response to parasitic infection, we suspected that a parasitic infection might be the cause of the increased tuft cells in the *WT* mouse line. We collected fecal samples from *WT* mice and extracted DNA, then used quantitative PCR to screen for parasitic infection. Similar to what has been found by others in bred-in-house mice^6^, we identified *Tritrichomonas muris* (Tm) in the fecal samples of *WT* mice (Fig. 1c).

To see whether Raptor deficiency affected tuft cells in the small intestine, *WT* and *i-Raptor−/−* mice were intraperitoneally injected with Tamoxifen (TAM) for 3 consecutive days and small intestinal tuft cells examined 14 days later. We found that tuft cells abundance was significantly less in i-Raptor −/− mice (Fig. 1a,b). Western blot assay using DCLK1 antibody confirmed the dramatic reduction of tuft cells in i-Raptor−/− mice (Fig. 1d). As previously reported^13^, the effective deletion of Raptor and mTORC1 inactivation in small intestinal epithelial cells by this strategy was also confirmed (Fig. 1d).

To further analyze whether Raptor deficiency-induced reduction of tuft cells affected the functional type 2 immune response, we measured IL-25, IL-33 and thymic stromal lymphopoietin (TSLP) expression in isolated intestinal epithelial cells as well as IL-13, IL-4 and IL-5 expression in the lamina propria, as these cytokines are thought to be involved in the parasite-induced type 2 immune response. IL-25 mRNA expression significantly decreased in i-Raptor−/− epithelial cells, TSLP expression is very low relative to IL-25, but deleting Raptor rendered it undetectable, while IL-33 expression was not affected by Raptor deficiency (Fig. 1e). IL-13 mRNA expression was significantly blunted within the underlying lamina propria in i-Raptor−/− mice, however, IL-4 and IL-5 expression was not significantly changed (Fig. 1f). Despite the dampened type 2 immune response in i-Raptor−/− mice, the Tm burden in those mice was only slightly increased. The slightly elevated Tm burden was not associated with Raptor deficiency since it was also manifested in WT mice after TAM treatment (Fig. 1g). These data is consistent with recent findings that parasites stimulates tuft cells to secrete IL-25 which in turn induces IL-13 expression in the lamina propria to initiate type 2 immune response in small intestine. It further indicates that epithelial cell specific Raptor is required for maintaining an active type 2 immune response to parasitic infection of the small intestine.

**Figure 2:**
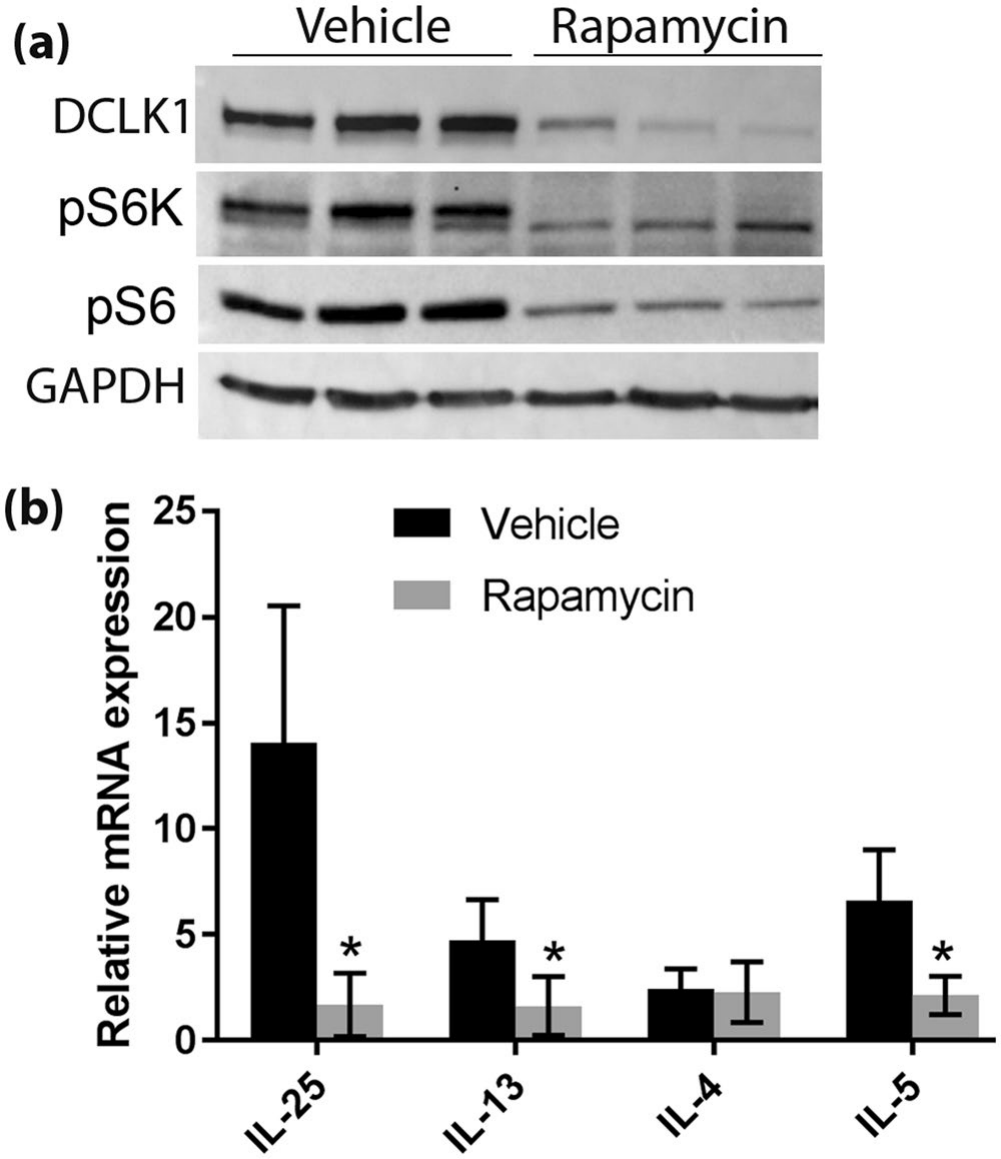
Systemic mTORC1 inhibition diminishes tuft cells. *Vilin-Cre* ^*ER*^ (+/−); *Raptor (f/f)* mice were randomized into 2 groups (n = 5 for Vehicle and n = 6 for Rapamycin) and injected intraperitoneally with either vehicle (5% Tween-80, 5% PEG-400 in PBS) or Rapamycin (4 mg/kg body weight) for 8 days before sacrifice. (**a**) Representative Western blot analysis of the protein expression in enterocytes as indicated. GAPDH was used as a loading control. (**b**) Real-time PCR quantification of IL-25 expression in enterocytes and IL-13/IL-4/IL-5 expression in lamina propria.

Raptor is a scaffold protein required for mTORC1 activation in intestinal epithelial cells. To test whether Raptor regulation of tuft cell abundance is dependent on its function on inactivating mTORC1, rapamycin was intraperitoneally injected into Tm infected WT mice for 8 days to inactivate mTORC1 signaling. The efficacy of Rapamycin was confirmed by decreased pS6K and pS6 phosphorylation in isolated enterocytes (Fig. 2a). Similar to what we have found in Raptor deficient enterocytes, treatment of Tm infected mice with Rapamycin resulted in reduced expression of DCLK1 and IL-25 in the small intestinal epithelium and IL-13 in the mesenchyme. However, IL-5 expression in the mesenchyme, which is not stimulated by IL-25 during typical type 2 immune response, was also inhibited by Rapamycin (Fig. 2b). These data suggest that the mechanism for Raptor regulation of the type 2 immune response most likely dependent of its function on inhibiting mTORC1 in intestinal epithelial cells.

### Diet affects Tm viability in small intestine

**Figure 3:**
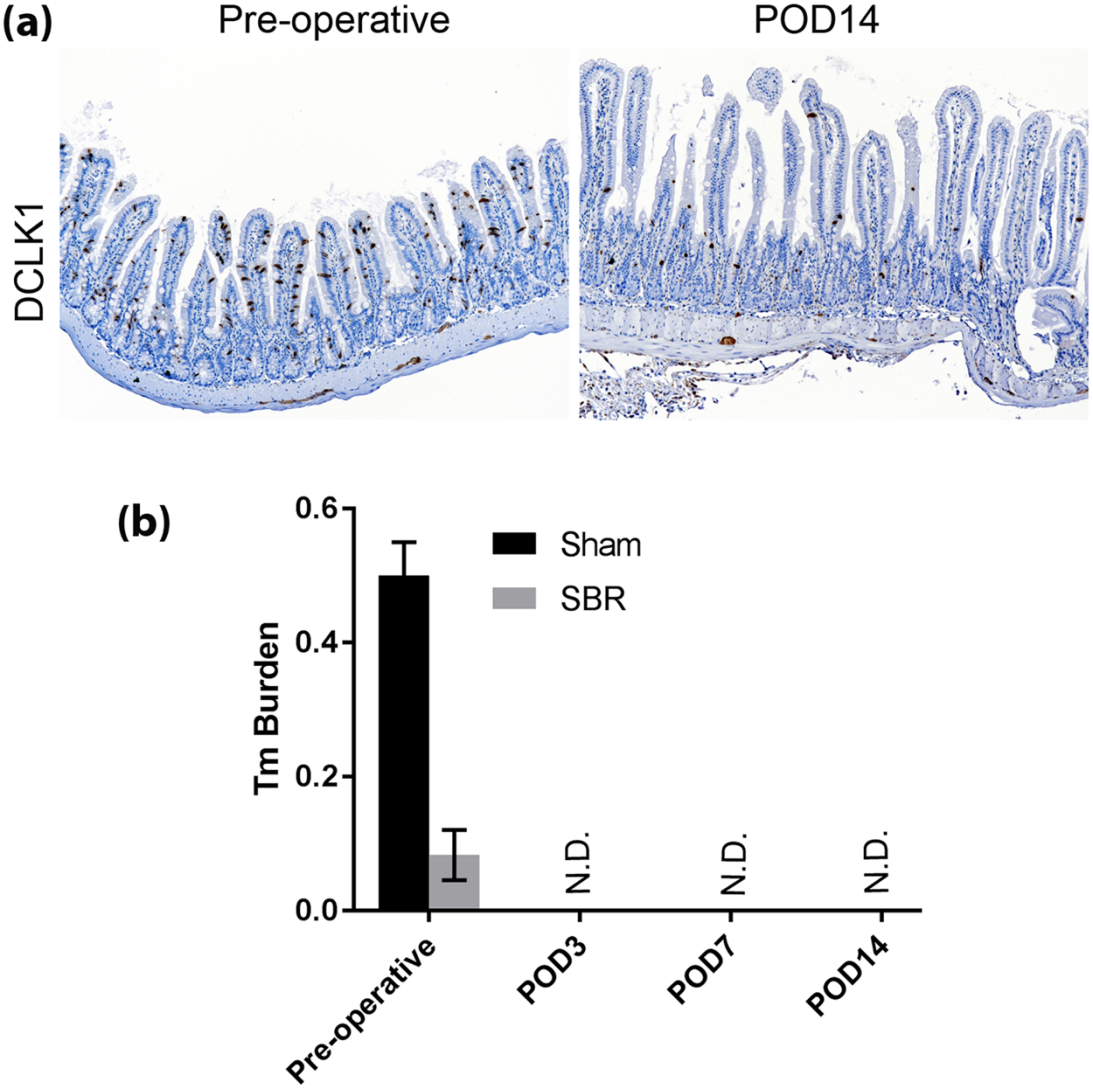
liquid diet is sufficient to eliminate Tm in mice. *Vilin-Cre* ^*ER*^ (−)*; Raptor (f/f)* (*WT*, n = 4 per group) mice were subject to 50% proximal SBR or Sham operation, small intestinal tissue and stool was collected at the time of surgery and different days after surgery as indicated. (**a**) Representative IHC shows tuft cells in brown using anti-DCLK1 antibody. POD14 indicates postoperative day 14. (**b**) Real-time PCR quantification of Tm burden in collected stool as indicated.

The microbiome of the small bowel is altered by small bowel resection (SBR) as we and others have reported previously^19, 20^. To better understand the effects of SBR on parasites community and the type 2 immune response, we performed 50% SBR on Tm infected WT mice. Small intestinal tissue and feces were collected before resection and 14 days post-resection. When compared to pre-operative tissue, DCLK1 positive tuft cells were drastically reduced in the postoperative small intestine as demonstrated by IHC staining (Fig. 3a). In addition, RT-PCR using Tm specific primers demonstrated a total loss of Tm (undetectable) after resection in the feces (Fig. 3b). To see whether the clearance of Tm was specific to SBR, we performed sham operations (intestinal transection and reanastomosis but without resection). Surprisingly, even the sham-operated WT mice cleared Tm in the feces 14 days after resection (Fig. 3b). Accordingly, IL-25 expression in epithelial cells and IL-13 expression in the mesenchyme was also lost in the postoperative small intestine (data not shown).

To investigate whether the clearance of Tm in postoperative mice was due to accelerated intestinal expulsion of Tm after surgery, we observed Tm at earlier time points after Sham or SBR surgery. Even though Tm burden was much higher in Sham group in this experiment, we found that Tm was already undetectable 3 days post-surgery in both operative groups (Fig. 3b). Since different operation types showed no difference in effectiveness in eliminating Tm, we decided to examine the effects of the standard liquid diet which was consumed by both groups after surgery. To this end, we simply treated un-operated Tm infected WT mice with liquid diet and collected feces before the treatment and 7 days after the treatment. We found that the liquid diet alone eliminated the Tm community (data not shown). The effectiveness of the feeding diet in eliminating Tm is compatible to the common anti-protozoal drug metronidazole, as Tm burden started to diminish 3 days after giving metronidazole in drinking water (data not shown). The unexpected findings with feeding diet demand more in-depth study, as they suggest that diet alone may be effective treatment for Tm infection and offer an alternative to anti-protozoal medication. Further investigation into the treatment of Tm is beyond of the scope of this study.

### Raptor is required for the initiation of a type 2 immune response to Tm infection

**Figure 4:**
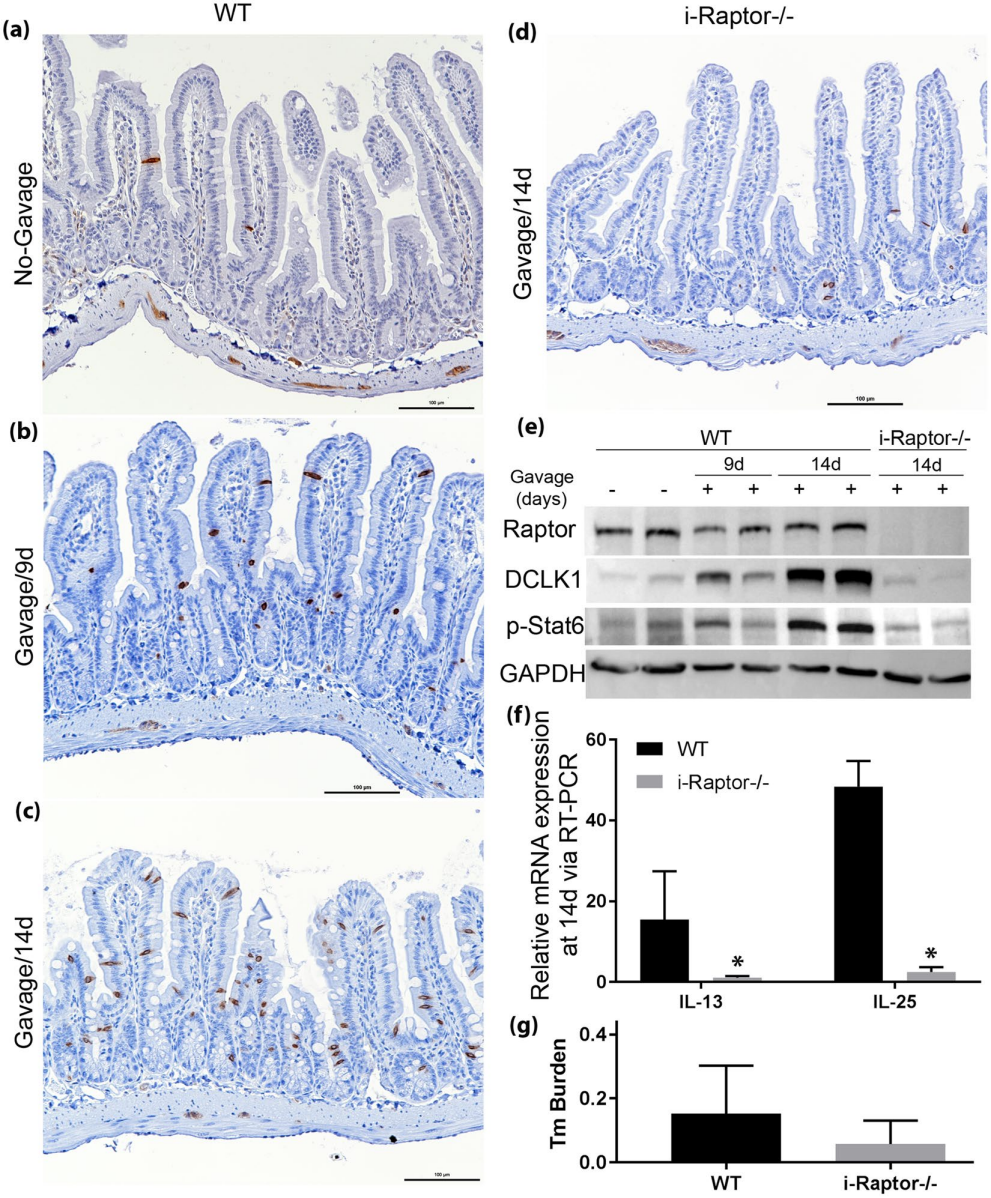
Raptor is required for initiating type 2 immune response after Tm infection. *Vilin-Cre* ^*ER*^ (−); *Raptor* (*f*/*f*) (WT, n = 6) and *Vilin-Cre* ^*ER*^ (+); *Raptor* (*f*/*f*) (*i*-*Raptor*−/−, n = 5) mice were injected intraperitoneally with TAM for 3 consecutive days, fed with liquid diet for 7 days to eliminate Tm infection and reinfected via gavage with Tm containing feces. The small intestine was removed at 9 or 14 days after gavage. (**a**–**d**) Representative IHC shows tuft cells in brown using anti-DCLK1 antibody. (**e**) Western blot analysis of DCLK1 protein expression and Stat 6 phosphorylation after Tm re-infection in mice. GAPDH was used as loading control. (**f**) Real-time PCR quantification of IL-25 expression in enterocytes and IL-13 expression in lamina propria. (**g**) Tm burden quantified from stools collected at 14 days after Tm gavage. Mean ± SD is shown. Student’s t test (*p < 0.05).

Our data has established that Raptor protein is indispensable for maintaining the type 2 immune response when mice are already infected with Tm. To further investigate whether epithelial Raptor is necessary for initiating the type 2 immune response to a fresh infection, we first treated the *WT* and *i-Raptor−/−* mice with liquid diet for 1 week effectively clearing the parasite. TAM was injected for 3 consecutive days and mice were rested for 7 days to allow deletion of Raptor in the epithelial cells. These mice were gavaged once with Tm containing feces and harvested at 9 and 14 days after the initial gavage. Fecal samples were collected after liquid diet to confirm clearance of the parasite and after gavage to confirm re-infection. All fecal samples from both WT and i-Raptor−/− mice tested positive for Tm and there was no difference in Tm burden at 14 days after gavage (Fig. 4g). However, only the WT mice manifested a typical type 2 immune response with elevated tuft cell numbers as demonstrated by the expression of DCLK1 in intestinal epithelial cells (Fig. 4a–c and e). Increased IL-25 expression in the epithelial cells and IL-13 expression in lamina propria was also confirmed in the Tm gavaged WT mice at 14 days post gavage (Fig. 4f). In sharp contrast, the i-Raptor−/− mice did not respond to Tm infection as demonstrated by the lack of DCLK1 expression and IL-25 in epithelium and IL-13 in lamina propria (Fig. 4e and f). Similar to what has been reported^6^, we found that Tm quickly colonized the mouse intestine and behaved like a commensal microbe. Contrary to intestinal worms, Tm could not be cleared by type 2 immune response in the small intestine. In summary, these data clearly indicate that epithelial Raptor is required for initiating type 2 immune response upon Tm infection.

### Stat 6 is phosphorylated in Tm infected intestinal epithelial cells independent of Raptor

**Figure 5:**
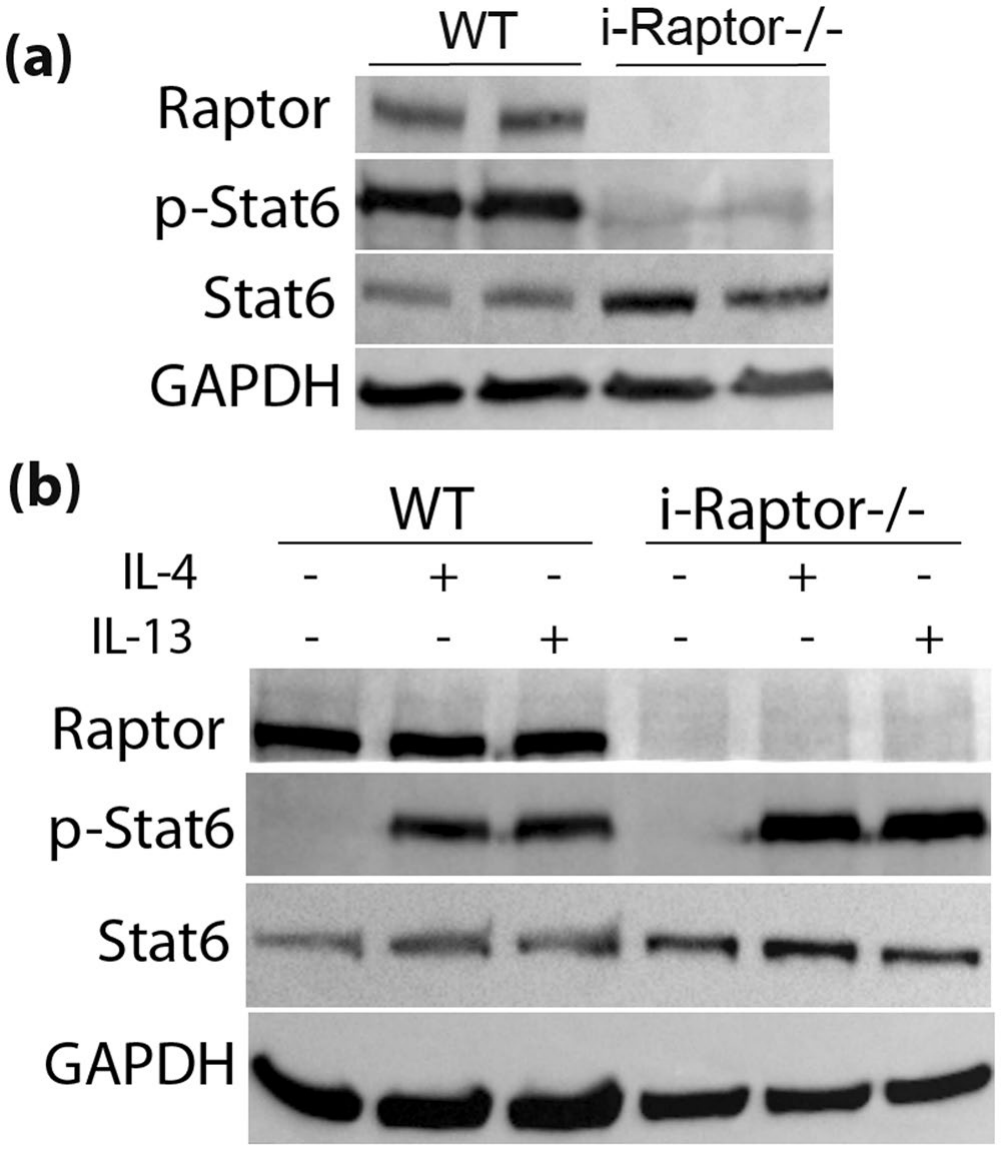
Raptor is dispensable for Stat 6 phosphorylation in Tm infected enterocytes. (**a**), *Vilin-Cre* ^*ER*^ (−); *Raptor* (*f*/*f*) (WT, n = 3) and *Vilin-Cre* ^*ER*^ (+); *Raptor* (*f*/*f*) (*i*-*Raptor*−/−, n = 3) mice were injected intraperitoneally with TAM for 3 consecutive days and small intestine removed 7 days later. (**a**) Representative Western blot analysis of Stat 6 expression and phosphorylation in enterocytes. (**b**) Enterocytes were isolated from the small intestine and incubated with vehicle, 50 ng/ml IL-4 or 50 ng/ml IL-13 for 10 minutes *in vitro*. Stat 6 activation was analyzed via Western blot.

To study the mechanism behind how Raptor might be regulating type 2 immunity, we investigated Stat 6. Stat 6 is activated by IL-4/IL-13 and has been reported as an essential protein for type 2 immune reactions in the intestine^6, 21^. We found that Stat 6 was phosphorylated in intestinal epithelial cells of the Tm infected WT mice and markedly diminished in the i-Raptor−/− epithelial cells (Fig. 5a). These results suggest that Raptor might be involved in regulating phosphorylation of Stat 6 in response to Tm infection. To test this hypothesis, we isolated intestinal epithelial cells from Tm-deprived WT and i-Raptor−/− mice, then stimulated them with IL-4 & IL-13 for 10 minutes *ex vivo* to induce Stat 6 phosphorylation. The intestinal epithelial cells responded to IL-4 & IL-13 with strong Stat 6 phosphorylation in both WT and i-Raptor−/− cells (Fig. 5b). This finding suggests that Raptor is not essential for Stat 6 phosphorylation in enterocytes. To further delineate why Stat 6 phosphorylation was absent in the enterocytes of i-Raptor−/− mice *in vivo*, we measured Stat 6 phosphorylation in the previously mentioned Tm gavaged mice. We found that Stat 6 was fully phosphorylated at day 14 in WT mice, but not i-Raptor−/− mice (Fig. 4e). These data suggest that the phosphorylation of Stat 6 in epithelial cells requires adequate IL-13/IL-4 levels whose production is dependent on the positive feedback of the type 2 immune response. In the absence of epithelial Raptor, a type 2 immune response could not be initiated and no IL-13 could be produced to activate Stat 6.

### Raptor is not required for tuft cell differentiation in intestinal organoid

**Figure 6:**
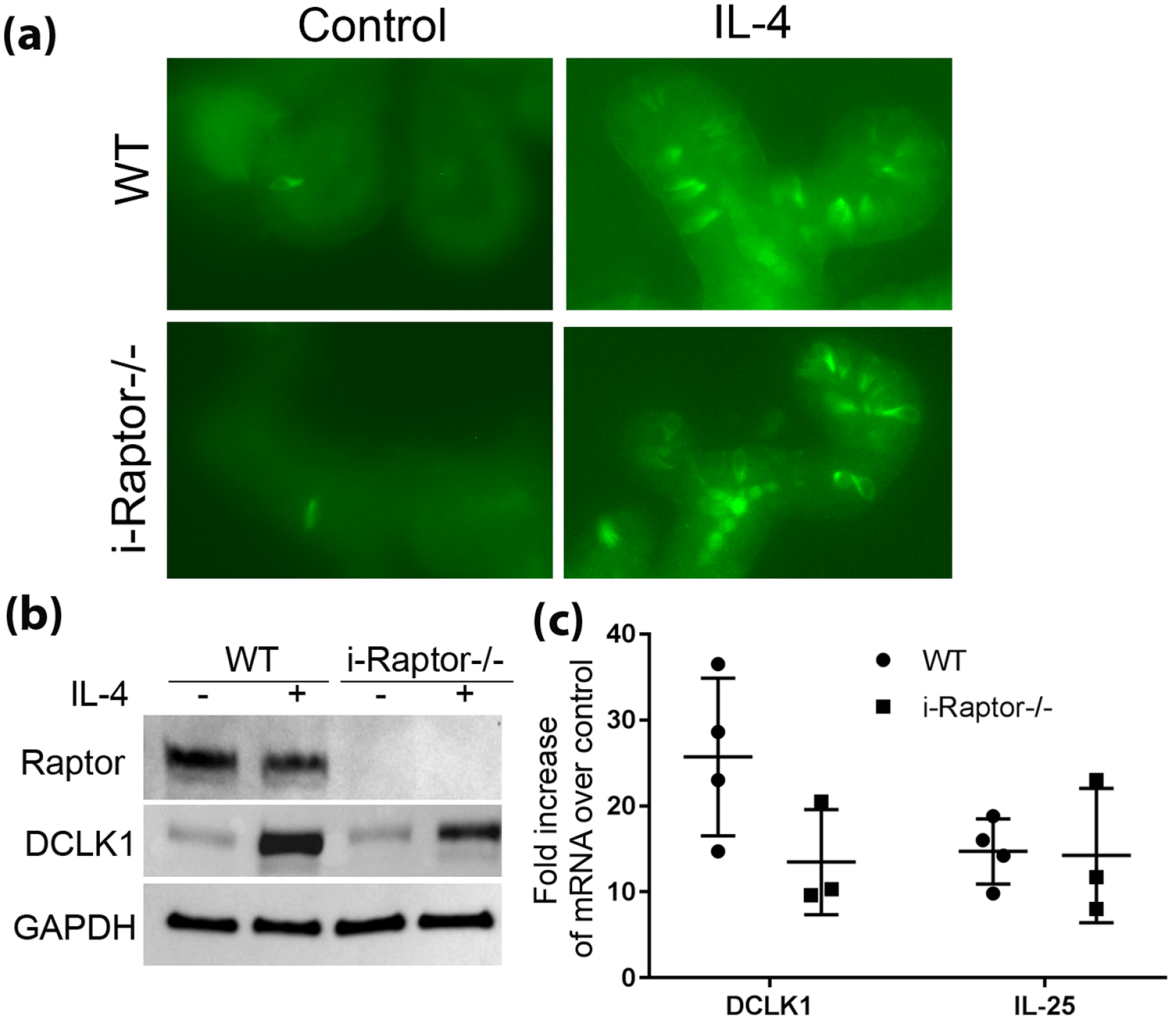
Raptor is not required for tuft cell differentiation in intestinal organoids. *Vilin-Cre* ^*ER*^ (−); *Raptor* (*f*/*f*) (WT, n = 3) and *Vilin-Cre* ^*ER*^ (+); *Raptor* (*f*/*f*) (*i*-*Raptor*−/−, n = 3) mice were injected intraperitoneally with TAM for 3 consecutive days, fed with liquid diet for 7 days and the small intestine removed at the end of the treatment. Crypts were isolated, mixed with matrigel and cultured into organoids. Organoids were then passaged and stimulated with 50 ng/ml IL-4 for 48 hours. (**a**) Representative whole-mount immunofluorescence shows tuft cells in green using DCLK1 antibody. (**b**) Western blot assay demonstrated DCLK1 expression after IL-4 stimulation. Raptor deletion in the organoid was also confirmed. GAPDH was used as loading control. (**c**) DCLK1 and IL-25 mRNA expression was measured via Real-time PCR assay.

Our next aim was to determine the mechanism behind the diminished DCLK1 positive tuft cells in i-Raptor−/− mice after Tm infection, specifically we wanted to explore the role of Raptor in tuft cell differentiation in the stem & transient amplifying cells. Since intestinal organoid alone could respond to IL-4 & IL-13 stimulation and differentiate equally into tufts^5^, we isolated crypts from the small intestine of WT or i-Raptor−/− mice and cultured them into organoids. When the organoids were stimulated with IL-4 for 48 hours, there was a dramatic increase in tuft cells in both WT and i-Raptor−/− organoids when detected by immunofluorescence using anti-DCLK1 antibody (Fig. 6a). When organoid protein expression was analyzed by Western blot, IL-4 stimulation resulted in a sharp increase in DCLK1 expression which appeared to be independent of Raptor protein (Fig. 6b). When DCLK1 transcription was measured by real-time PCR, no significant difference was found between Raptor deficient and WT organoid (Fig. 6c). Furthermore, IL-25, which is exclusively expressed by tuft cells, was greatly enhanced in response to IL-4 stimulation in both WT and Raptor deficient organoid (Fig. 6c). The limited effects of Raptor on tuft cell differentiation strongly suggests that Raptor’s main function is involved with the relay of signals from enterocytes to immune cells after parasitic infection in order to initiate the type 2 immune response.

## Discussion

Tuft cells are not a newly discovered cell type, but their function was poorly understood until recent. It appears that tuft cells can act as a reserve stem cell population^3^ and colon cancer-initiating cells^4^. They have been shown to enhance epithelial integrity and are essential for protecting the host during enteric infections and inflammatory reactions^22, 23^. Recently, a positive feed-back loop between tuft cells, immune-cells and epithelial progenitor/stem cells in response to parasitic infection was discovered^5–7^. How tuft cells sense the signals from parasitic infection to initiate the production of IL-25 is still unclear. However, in this process, taste receptor Trpm 5, G protein alpha-gustducin, transcription factor Pou2f3 as well as other factors have been found to be critical.

We found that a liquid rodent diet effectively treated Tm infection. To our knowledge, this is the first time that diet alone has been demonstrated to mitigate a parasitic infection. The liquid diet that was employed in this study has been standard for our laboratory for feeding mice that undergo intestinal anastomosis, since it significantly reduces postoperative bowel obstruction. This formulation consists of 17.3% protein/ 35% fat/ 47.7% carbohydrates and is slightly higher in fat content than standard rodent diet. Dietary fat has been suggested to regulate intestinal immune system^24^ and a recent study found that dietary fat could influence immune system via changing T cell membrane structure^25^. Whether the feeding diet used in our mouse surgical model activates intestinal immune system to actively kill Tm deserves further examination.

In our study, we used a conditional and inducible strategy to achieve targeted deletion of an important mTORC1 scaffold protein, Raptor, in the intestinal epithelium to study tuft cell differentiation. The mouse line we generated was incidentally contaminated with Tm and showed a strong type 2 immune response with elevated tuft cell numbers which was blunted in the absence of epithelial cell specific Raptor. The diminished tuft cells seen in Raptor null mice is unlikely caused by decreased proliferation, as our lab and others have found that Raptor deficiency does not affect crypt proliferation^13, 14^. However, Raptor deficiency does affect secretory cell linage differentiation in the small intestine as revealed by recent studies^13, 14^. It is especially intriguing when considering the effect of Raptor on Goblet cell differentiation since parasites also stimulate Goblet cell hyperplasia. Whether the reduced Goblet cells in Raptor deficient mice is due to Raptor itself or Raptor mediate type 2 immune response needs further investigation.

It is highly tempting to suggest that Raptor may regulate type 2 immune response by affecting stem cell differentiation which is a critical step in the type 2 immune response positive feedback loop. However, our organoid stimulation data suggested that this might not be the case. In the absence of Raptor, tuft cells could still be induced from organoids when stimulated with IL-4. Since epithelial Raptor is required for generating a type 2 immune response, our organoid culture results strongly suggest that epithelial Raptor likely functions by relaying signals of a parasitic infection from enterocytes to the underlying immune cells to induce tuft cell hyperplasia. The exact mechanism, however, still needs further *in vivo* investigation and remains unresolved.

The function of Raptor in this process seems to be dependent on its role as an inhibitor of mTORC1 activity. How exactly mTORC1/Raptor integrates with other factors such as Trpm 5, G protein alpha-gustducin, transcription factor Pou2f3 or other unknown players to initiate type 2 immune response requires further investigation. Nonetheless, the mTOR pathway has been considered as a master regulator of immune responses both in adaptive and innate immunity^26, 27^. In this study, we used a conditional and inducible strategy to inactivate mTORC1 specifically in intestinal mucosa and discovered that the mTORC1 function in the epithelial cells rather than the immune cells plays a key role in the type 2 immune response to parasitic infection. It has been reported that tuft cells are also the primary source of IL-25 in the lung^7^ which may cause airway hyperresponsiveness in allergic asthma^28^. We have demonstrated that targeting mTORC1 in the epithelial cell alone may prevent a type 2 immune response, thus providing a new strategy for treating allergic induced hyperactive type 2 immune reaction.
